# Outcome differences in HPV-driven head and neck squamous cell carcinoma attributable to altered human leukocyte antigen frequencies

**DOI:** 10.3389/fonc.2023.1212454

**Published:** 2023-12-21

**Authors:** Gunnar Wichmann, Nathalie Vetter, Claudia Lehmann, Ramona Landgraf, Ilias Doxiadis, Rebecca Großmann, Ekaterina Vorobeva, Andreas Dietz, Veit Zebralla, Susanne Wiegand, Theresa Wald

**Affiliations:** ^1^ Department of Otorhinolaryngology, Head and Neck surgery, University Hospital Leipzig, Leipzig, Germany; ^2^ Institute for Transfusion Medicine, Transplantation Immunology, University Hospital Leipzig, Leipzig, Germany

**Keywords:** human papillomavirus (HPV), oropharyngeal squamous cell carcinoma (OPSCC), head and neck cancer, outcome research, human leukocyte antigen (HLA), haplotype, Genetic association, progression-free survival (PFS)

## Abstract

**Background:**

Effective immune surveillance requires a functioning immune system and natural killer (NK) and T cells for adequate innate and antigen-specific immune responses critically depending on human leukocyte antigens (HLAs) and haplotypes representing advantageous combinations of HLA antigens. Recently, we reported a link between altered frequencies of HLA alleles and haplotypes and developing head and neck squamous cell carcinoma (HNSCC). Whereas the majority of HNSCCs seem to be related to classical risk factors alcohol and tobacco, a subset of HNSCC and especially oropharyngeal squamous cell carcinoma (OPSCC) were etiologically linked to human papillomavirus (HPV) recently. Here, we demonstrate in HPV-driven (p16-positive high risk-HPV DNA-positive) HNSCC a deviating distribution of HLA antigens and haplotypes and their relevance to outcome.

**Methods:**

Leukocyte DNA of *n* = 94 HPV-driven HNSCC patients (*n* = 57 OPSCC, *n* = 37 outside oropharynx) underwent HLA SSO typing, allowing allele, antigen (allele group), and haplo-typing. Besides comparing these frequencies with those of German blood donors, we analyzed their impact on outcome using Kaplan–Meier plots and Cox proportional hazard regression.

**Results:**

Antigen and haplotype frequencies demonstrate enrichment of rare antigens and haplotypes. The HLA score for unselected HNSCC patients was not predictive for outcome here. However, together with alcohol consumption, tobacco smoking, T category, and extranodal extension of locoregional metastases and treatment applied, eight HLA traits allow for predicting progression-free and tumor-specific survival.

**Conclusion:**

Patients can be categorized into low, intermediate-low, intermediate-high, and high risk groups. Using a new PFS risk score for HPV-driven HNSCC may allow to improve prognostication.

## Introduction

Effective immune surveillance requires a functioning immune system and natural killer (NK) and T cells for adequate innate and antigen-specific immune responses. These critically depend on proteins encoded by genes of the major histocompatibility complex, human leukocyte antigen (HLA) alleles on chromosome 6 in man. As loci of class I (HLA-A, -B, and -C) and II (HLA-DP, -DQ, and -DR) are in near proximity, the polymorphic alleles are mostly inherited combined in blocks of genes, so-called haplotypes representing advantageous combinations of HLA antigens able to present an overlapping broad spectrum of peptides derived from various antigens including tumor-associated antigens (TAAs) for presentation to antigen-specific T cells, while also maintaining interaction with receptors of NK cells (1–10). As we recently could demonstrate a link between altered HLA allele and haplotype frequencies in head and neck squamous cell carcinoma (HNSCC) (11) and developed an HLA score for HNSCC related to classical risk factors alcohol and tobacco (12), we were interested if these findings and the predictive value of the HLA score could be replicated in HPV-driven (p16-positive high-risk HPV DNA-positive) oropharyngeal squamous cell carcinoma (OPSCC) and other HPV-driven HNSCCs arising outside the oropharynx or if other HLA traits could be involved in development and are predictive for outcome in this subgroup of HNSCC. Taking into account not only the clinical characteristics of the tumor and the patient but also lifestyle-associated risk factors, we provide a newly developed score based on a multivariate Cox proportional hazard regression model for progression-free survival (PFS) that predominantly depends on genetic information, HLA antigens, and haplotypes in particular.

## Materials and methods

### Patients and pathologic tumor data

This study was carried out in accordance with the recommendations of the guidelines of the ethics committee of the Medical Faculty of the University Leipzig. The protocol was approved by the ethics committee of the Medical Faculty of the University Leipzig (vote no. 201-10-12072010, no. 202-10-12072010, and no. 341-15-09102015). All subjects gave written informed consent in accordance with the Declaration of Helsinki. Patho-histological characteristics including ENE (+/−) and epidemiological risk factors (alcohol and tobacco smoking history) were recorded.

### Clinical workup for HNSCC

All patients underwent standardized diagnostics according to national (German) and NCCN guidelines (13) and were staged according to TNM 7th edition (14) and treated as consented in the multidisciplinary tumor board of our certified head and neck cancer center according to standardized operating procedures in agreement with national and international guidelines as published earlier (15–19). HPV genotyping and p16 immune histochemistry were executed as reported previously (19), and only HPV-driven cases were enrolled in the study. To show TNM staging according to the current TNM nomenclature, TNM categories and stages after re-staging according to TNM 8th edition (20) are shown in **Table 1**.

**Table 1:** Distribution of characteristics among *N* = 94 patients with HPV-driven (HPV-DNA+ RNA+ or HPV-DNA+ p16+) head and neck squamous cell carcinoma with primary either in the oropharynx (OPSCC; *n* = 57, 60.6%) or outside the oropharynx (other; *n* = 37, 39.4%); the respective odds ratio (OR) and 95% confidence interval (95% CI) are accompanied by the two-sided *p*-value.

	Total		OPSCC		Other HNSCC			
	*n*	(%)	*n*	(%)	*n*	(%)	OR (95% CI)	*p*-value ^†^
Sex								
Female	24	(25.5)	12	(21.1)	12	(32.4)	Ref. (0.323 –3.101)	0.216
Male	70	(74.5)	45	(78.9)	25	(67.6)	1.8 (0.705 –4.597)	
Age Score								
< 50 years	11	(11.7)	5	(8.8)	6	(16.2)	Ref. (0.187 –5.357)	0.415
50 –< 60 years	34	(36.2)	20	(35.1)	14	(37.8)	1.714 (0.436 –6.742)	
60 –< 70 years	34	(36.2)	24	(42.1)	10	(27.0)	2.880 (0.712 –11.65)	
≥ 70 years	15	(16.0)	8	(14.0)	7	(18.9)	1.371 (0.288 –6.535)	
Smoker status								
Never	20	(21.3)	10	(17.5)	10	(27.0)	Ref. (0.289 –3.454)	0.487
Former	16	(17.0)	11	(19.3)	5	(13.5)	2.200 (0.557 –8.686)	
Current	58	(61.7)	36	(63.2)	22	(59.5)	1.636 (0.587 –4.559)	
Smoker category								
Never smoker	20	(21.3)	10	(17.5)	10	(27.0)	Ref. (0.289 –3.454)	0.332
< 10 pack years	8	(8.5)	5	(8.8)	3	(8.1)	1.667 (0.311 –8.929)	
10–< 20 pack years	15	(16.0)	8	(14.0)	7	(18.9)	1.143 (0.299 –4.367)	
20–< 30 pack years	20	(21.3)	17	(29.8)	3	(8.1)	5.667 (1.254 –25.61)	
30–< 40 pack years	17	(18.1)	9	(15.8)	8	(21.6)	1.125 (0.308 –4.105)	
40–< 50 pack years	10	(10.6)	6	(10.5)	4	(10.8)	1.500 (0.322 –6.991)	
≥ 50 pack years	4	(4.3)	2	(3.5)	2	(5.4)	1.000 (0.117 –8.560)	
Alcohol category								
never	18	(19.1)	12	(21.1)	6	(16.2)	Ref. (0.250 –3.999)	0.930
1–30 g/d	46	(48.9)	27	(47.4)	19	(51.4)	0.711 (0.227 –2.227)	
31–60 g/d	11	(11.7)	7	(12.3)	4	(10.8)	0.875 (0.182 –4.212)	
> 60 g/d	19	(20.2)	11	(19.3)	8	(21.6)	0.688 (0.180 –2.620)	
T category 7th edition^‡^								
T1	17	(18.1)	11	(19.3)	6	(16.2)	Ref. (0.245 –4.083)	0.782
T2	28	(29.8)	17	(29.8)	11	(29.7)	0.843 (0.241 –2.945)	
T3	22	(23.4)	12	(21.1)	10	(27.0)	0.655 (0.178 –2.405)	
T4a	24	(25.5)	16	(28.1)	8	(21.6)	1.091 (0.295 –4.033)	
T4b	2	(2.1)	1	(1.8)	1	(2.7)	0.545 (0.029 –10.37)	
Tx	1	(1.1)	0	–	1	(2.7)	0.188 (0.007 –5.328) ^#^	
T category 8th edition^‡‡^								
T1	17	(18.1)	11	(19.3)	6	(16.2)	Ref. (0.245 –4.083)	**< 0.001**
T2	28	(29.8)	17	(29.8)	11	(29.7)	0.843 (0.241 –2.945)	
T3	22	(23.4)	12	(21.1)	10	(27.0)	0.655 (0.178 –2.405)	
T4	17	(18.1)	17	(29.8)	0	–	19.78 (1.014 –386.0) ^#^	
T4a	8	(8.5)	0	–	8	(21.6)	0.033 (0.002 –0.675) ^#^	
T4b	1	(1.1)	0	–	1	(2.7)	0.188 (0.007 –5.328) ^#^	
T4x	1	(1.1)	0	–	1	(2.7)	0.188 (0.007 –5.328) ^#^	
N category 7th edition^‡^								
N0	33	(35.1)	13	(22.8)	20	(54.1)	Ref. (0.373 –2.685)	**0.014**
N1	17	(18.1)	13	(22.8)	4	(10.8)	5.000 (1.335 –18.72)	
N2a	5	(5.3)	5	(8.8)	0	–	16.70 (0.852 –327.4) ^#^	
N2b	16	(17.0)	13	(22.8)	3	(8.1)	6.667 (1.585 –28.04)	
N2c	11	(11.7)	6	(10.5)	5	(13.5)	1.846 (0.466 –7.316)	
N3	12	(12.8)	7	(12.3)	5	(13.5)	2.154 (0.562 –8.254)	
N category 8th edition^‡‡^								
N0	33	(35.1)	13	(22.8)	20	(54.1)	Ref. (0.373 –2.685)	**< 0.001**
N1	37	(39.4)	33	(57.9)	4	(10.8)	12.69 (3.634 –44.33)	
N2	11	(11.7)	11	(19.3)	0	–	34.93 (1.896 –643.5) ^#^	
N2b	1	(1.1)	0	–	1	(2.7)	0.506 (0.019 –13.37) ^#^	
N2c	3	(3.2)	0	–	3	(8.1)	0.217 (0.010 –4.543) ^#^	
N3b	9	(9.6)	0	–	9	(24.3)	0.080 (0.004 –1.490) ^#^	
UICC 7th edition^‡^								
I	7	(7.4)	2	(3.5)	5	(13.5)	Ref. (0.098 –10.17)	0.055
II	10	(10.6)	3	(5.3)	7	(18.9)	1.071 (0.128 –8.977)	
III	17	(18.1)	12	(21.1)	5	(13.5)	6.000 (0.859 –41.90)	
IVA	46	(48.9)	32	(56.1)	14	(37.8)	5.714 (0.987 –33.08)	
IVB	14	(14.9)	8	(14.0)	6	(16.2)	3.333 (0.473 –23.47)	
UICC 8th edition^‡‡^								
I	29	(30.9)	24	(42.1)	5	(13.5)	Ref. (0.256 –3.906)	**< 0.001**
II	19	(20.2)	12	(21.1)	7	(18.9)	0.357 (0.093 –1.365)	
III	27	(28.7)	21	(36.8)	6	(16.2)	0.729 (0.194 –2.739)	
IVA	10	(10.6)	0	–	10	(27.0)	0.011 (0.001 –0.211) ^#^	
IVB	9	(9.6)	0	–	9	(24.3)	0.012 (0.001 –0.235) ^#^	
ENE ^§^								
0	26	(27.7)	19	(33.3)	7	(18.9)	Ref. (0.294 –3.406)	**0.008**
1	35	(37.2)	25	(43.9)	10	(27.0)	0.912 (0.296 –2.866)	
Not applicable (N0)	33	(35.1)	13	(22.8)	20	(54.1)	0.239 (0.079 –0.729)	
Surgery								
No surgery	13	(13.8)	8	(14.0)	5	(13.5)	Ref. (0.206 –4.856)	0.943
Surgery	81	(86.2)	49	(86.0)	32	(86.5)	0.957 (0.287 –3.187)	
Radiotherapy								
No RT	25	(26.6)	11	(19.3)	14	(37.8)	Ref. (0.327 –3.055)	**0.047**
RT	69	(73.4)	46	(80.7)	23	(62.2)	2.545 (0.999 –6.484)	
Cisplatin								
No cisplatin	51	(54.3)	27	(47.4)	24	(64.9)	Ref. (0.459 –2.176)	0.096
Cisplatin	43	(45.7)	30	(52.6)	13	(35.1)	2.051 (0.875 –4.809)	
Chemo- and radiotherapy								
No CRT	86	(91.5)	51	(89.5)	35	(94.6)	Ref. (0.544 –1.838)	0.385
CRT	8	(8.5)	6	(10.5)	2	(5.4)	2.059 (0.393 –10.80)	
Operation + postoperative radiotherapy								
No Op+PORT	70	(74.5)	42	(73.7)	28	(75.7)	Ref. (0.509 –1.967)	0.829
Op+PORT	24	(25.5)	15	(26.3)	9	(24.3)	1.111 (0.428 –2.887)	
Bimodal therapy								
No Op+POR(C)T	36	(38.3)	18	(31.6)	18	(48.6)	Ref. (0.397 –2.519)	0.096
Op+PORT or Op+PORCT	58	(61.7)	39	(68.4)	19	(51.4)	2.053 (0.875 –4.817)	
Trimodal therapy								
Other	60	(63.8)	33	(57.9)	27	(73.0)	Ref. (0.487 –2.053)	0.137
Op-PORCT	34	(36.2)	24	(42.1)	10	(27.0)	1.964 (0.802 –4.811)	

^†^ p-values from Pearson’s Chi-square (χ^2^) tests for contingency tables; ^‡^ TNM staging according to 7th ed. 2010 (14); ^‡‡^ TNM staging according to 8th ed. 2017 (20); ^§^ ENE, extranodal extension; ^#^ corrected according to Cox to prevent division by zero (21, 22); significant p-values < 0.05 highlighted in bold.

### HLA typing

DNA samples from peripheral blood of 94 patients with HPV-driven HNSCC (19), *n* = 57 OPSCC and *n* = 37 with primary lesion outside the oropharynx and treated between 2012 and 2018, underwent low-resolution HLA typing utilizing OneLambda (West Hills, CA) SSO-typing kits for HLA-A, -B, -C, -DQ, and -DR according to the manufacturer’s instructions. Patterns of amplified transcripts/positive beads were used for software-based low- to medium-resolution typing of alleles and presence/absence of particular antigens (according to serologic epitopes/markers) using the HLA Fusion™ software (One Lambda, West Hills, CA). This allowed for the interpretation of the typing results regarding the presence of particular antigens encoded by the respective alleles.

### Determination of HLA haplotypes

As typing of parents and/or children of patients was impossible due to the patients*’* age, haplotypes were assessed as estimated haplotypes according to binary (exact) probabilities for joint presence of antigens (11, 12).

### Assessment of linkage disequilibrium

The absolute linkage disequilibrium *D_ab_* of antigens *a* and *b* in diplotypes was calculated as *D_ab_* = *f_ab_* − *f_a_f_b_*, and in triplotypes as *D_abc_* = *f_abc_* – *f_a_f_bc_* for antigen *a* and diplotype *bc* (23). The relative linkage disequilibrium *D_ab (rel)_* was calculated accordingly as *D_ab (rel)_* = *D_ab_*/*f_a_f_b_* for *D_ab_* < 0, *D _ab (rel)_* = *D_ab_*/*f_a_(*1 − *f_b_)* for *D_ab_* ≥ 0, if *f_b_* > *f_a_*, or *D_ab (rel)_* = *D_ab_*/*f_b_(*1 *− f_a_)*, if *D_ab_* ≥ 0, *f_a_* > *f_b_*. We calculated the expected frequency of the haplotype relative to antigen frequency in healthy adults and detected in our cohort and report the delta in haplotype frequencies as the difference between *D_ab (rel) OPSCC_* and *D_ab (rel) healthy_* as well.

### Statistical analysis

Statistical analyses using SPSS version 29 (24) included Pearson’s Chi-square (*χ* ^2^) tests to assess differences between categorical variables and distribution of antigens and haplotypes among patients. Benjamini–Hochberg correction (25) was used to correct for multiple testing, and the false-positive reporting probability (FPRP) was used to assess the noteworthiness of findings according to Wacholder et al. (26). Time-dependent covariates were measured from date of diagnosis to date of event. They included overall survival (OS; the time span from diagnosis until death of any cause by censoring patients alive at end of follow-up), tumor-specific survival (TSS; the time span from diagnosis until cancer-related death censoring patients alive at the end of follow-up or death from other causes), and PFS. PFS was defined as the time span from diagnosis until relapse or cancer-related death censoring patients alive at the end of follow-up. We also estimated local recurrence-free survival (LRFS), nodal recurrence-free survival (NRFS), locoregional (local + nodal) recurrence-free survival (LRRFS), and distant metastasis-free survival (DMFS), and second malignancy-free survival (SMFS) defined as the time span from diagnosis until the specific type of relapse was diagnosed by censoring patients alive without that specific type of relapse at the end of follow-up. Outcome differences between groups were analyzed using Kaplan–Meier cumulative survival plots and log-rank tests (27, 28). Univariate and multivariate Cox regression models (21, 22) were utilized to estimate each covariate’s hazard ratio (HR) and to identify independent predictors (*Pi*) of PFS among clinical characteristics and HLA antigens and haplotypes. To this end, we utilized the stepwise forward likelihood ratio function for Cox proportional hazard regression of SPSS 29 (24) for data-driven covariate extraction. The stability of the PFS model and the *Pi* identified was proven applying a bootstrap utilizing 1,000 iterations (29). The HR of each *Pi* was transformed into its natural logarithm (*ln _HR_*) and the sum of all *ln* values used to calculate each patient’s individual risk score. Using the 16th, 50th, and 84th percentile of PFS risk scores as cutoff, we categorized patients into low, intermediate-low, intermediate-high, and high risk (30, 31). *p*-values below 0.05 in two-sided tests were considered as significant.

## Results

Until the predefined inclusion limit of *n* = 37 cases with HPV-driven (19) HNSCC localized outside the oropharynx was achieved, a total sample of 94 patients were included in this study (**Table 1**). Contrasting the two groups of patients with HPV-driven cancer, *n* = 57 with OPSCC and *n* = 37 patients with HNSCC outside the oropharynx, characteristics such as sex, age at diagnosis, or classical risk factors for HNSCC like tobacco smoking or drinking alcohol (smoker and alcohol category) did not differ significantly between groups. However, statistically significant differences in the following clinical categories were observed: T category 8th edition (*p* < 0.001), N category 7th and 8th edition (*p* = 0.014 and *p* < 0.001), UICC 8th edition (*p* < 0.001), extranodal (aka extracapsular) extension (*p* = 0.017), and treatment including radiotherapy (*p* = 0.047).

**Figure 1:**
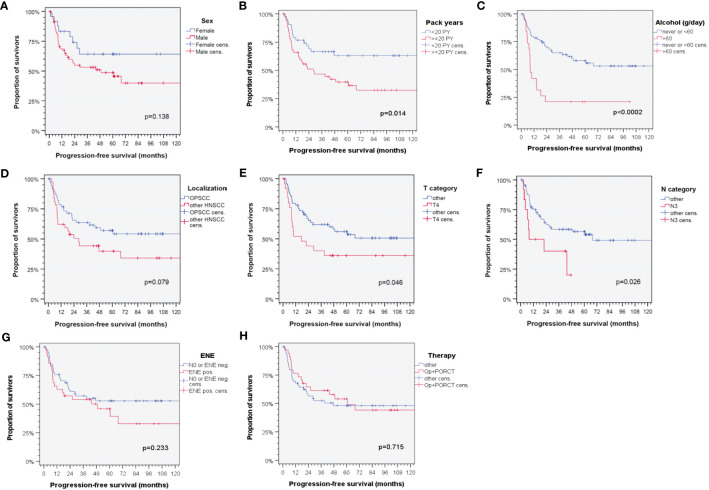
Kaplan–Meier cumulative survival plots (27) for progression-free survival of HPV-driven HNSCC for selected clinical characteristics with known impact on outcome in HNSCC based on literature data according to **(A)** sex; **(B)** tobacco smoking history (< 20 versus ≥ 20 pack years); **(C)** daily alcohol consumption (< 60 versus ≥ 60 g); **(D)** localization of the primary lesion within the oropharynx (ICD-10-C01, C05, C09, and C10) versus outside the oropharynx (ICD-10-C02, C04, C13, C30, C31, and C32) (14, 20); **(E)** T category [T4 versus other according to TNM 7th edition (14)]; **(F)** N category [N3 versus other according to TNM 7th edition (14)]; **(G)** extranodal extension of neck nodes (ENE versus no ENE or N0); and **(H)** applied therapy regimen (surgery followed by cisplatin-based postoperative radio-chemotherapy, Op+PORCT versus other therapy regimens). *p*-values shown are from log-rank tests.

Analyzing the impact of clinical and epidemiologic risk factors regarding PFS of patients, significant differences were observed according to smoking history (< 20 versus ≥ 20 pack years; *p* = 0.014), alcohol consumption (< 60 versus ≥ 60 g/day; *p* < 0.0002), T category (T4 versus other according to TNM 7^th^ edition; *p* = 0.046), and N category (N3 versus other according to TNM 7th edition; *p* = 0.026). Interestingly, some well-known risk factors failed in univariate analyses to demonstrate a significant impact on PFS in this cohort. These risk factors included sex, localization of the primary, extranodal extension, and therapy (**Figure 1**).

According to low-resolution HLA-typing results, we checked if the distribution of HLA traits reported to predict outcome in HNSCC and defining the earlier described HLA score (HLA-B13, -B35, -B51, -DQB1*06, homozygous C, homozygous -DRB4, haplotypes A1-B8 and B8-Cw7 with score 2, 1, 2, 1, 1, 2, -6, and 4) (12) are present in the same frequency in patients with HPV-driven HNSCC. We observed no significant differences in frequencies of each of the HLA score components (all *p* > 0.18). Summarizing them as HLA score, there was also no difference (*p* = 0.185). Focusing on HLA antigens, we found only A11 demonstrating significantly deviating frequency between HPV-driven HNSCC groups among patients of our cohort. However, some HLA haplotypes (diplo- or triplotypes) were significantly decreased compared to healthy German blood donors (**Table 2**). These were predominantly those with the highest prevalence in blood donors (A2-B62, A1-B8, B44-DR7, B62-DR4, A1-B8-DR3, A2-B7-DR15, A2-B60-DR13, A2-B62-DR4, A3-B35-DR1, and A29-B44-DR7) according to Müller et al. (23). Besides deviating haplotype frequencies, we also detected a lowered linkage disequilibrium in these haplotypes (**Table 2**) probably linked to disruption of haplotypes as recently described (11, 12). Moreover, carriers of the most prevalent haplotypes in healthy adults (23) demonstrated a reduced relative risk for HPV-driven HNSCC. In sharp contrast, we found increased frequencies of A3-B18, A3-B8, A1-B51, and A3-B51 in HPV-driven HNSCC, all representing haplotypes, which, in healthy blood donors, are among the rather seldom detected. This is reflected by an increased relative risk for the carriers of these haplotypes and especially the A3 haplotypes A3-B18 and A3-B8 (here often detected in unusual DR antigen combinations (23); **Table 2**). The increased prevalence of A24-B8-DR3 in eight patients (OR 12.4, 95% CI 0.99 –153.8; *p* = 0.051) is an example for such unusual haplotypes. A3 triplotypes rarely detected in blood donors demonstrated a significant higher frequency in our cohort (**Table 2**). However, significant differences in haplotype frequencies related to location of the primary lesion were not observed. The only exception was A3-B18.

**Table 2:** Frequencies and linkage disequilibrium (*D*) and relative linkage disequilibrium (*D_rel_*) in a large German sample of healthy blood donors (23) and HPV-driven head and neck squamous cell carcinoma (HNSCC; this study) accompanied by expected frequencies of HLA diplotypes and triplotypes significantly deviating from antigen-based haplotype frequencies in blood donors (23) and odds ratio (OR) and 95% confidence interval (95% CI) as well as two-sided *p*-value (*p*-value **^†^**), Benjamini–Hochberg corrected *p*-value (*p*-value **^††^**) (25), and false-positive reporting probability (FPRP) (26) in HPV-driven HNSCC patients.

	*f _observed_ ^I^*	*D_ab_ ^I^*	*D_ab_^(rel) I^*	*f _observed_* rel. to *f _expected_ ^I^*	*D_ab_ = f_ab_ -f_a_f_b_ ^II^*	*D_ab_^(rel) II^*	*f _observed_* rel. to *f _expected_* in HNSCC *^II^*	*Δ f _observed_* in HNSCC	*RR _diplotype carrier_*	*RR _diplotype non-carrier_*	*f _observed_* in HNSCC *n (%)*	*f _expected_ ^|^* in healthy adults *n (%)*	OR (95% CI)	*p-*value ^†^	*p-*value ^††^	FPRP level ^‡^
A2-B62	0.042	0.019	0.172	1.819	−0.0005	−0.0408	0.9592	−0.8601	0.2515	3.9762	4 (1.1)	15.9 (4.2)	0.243 (0.081 –0.736)	**0.0123**	>0.999	0.3573
A1-B8	0.083	0.066	0.519	4.925	0.0227	0.1925	2.5653	−2.3599	0.4489	2.2278	14 (3.7)	31.2 (8.3)	0.428 (0.224 –0.817)	**0.0101**	>0.999	0.1828
B44-DR7	0.030	0.015	0.105	1.954	−0.0005	−0.0600	0.9400	−1.0141	0.2662	3.7562	3 (0.8)	11.3 (3.0)	0.260 (0.072 –0.938)	**0.0396**	>0.999	0.7191
B62-DR4	0.029	0.019	0.209	2.805	0.0023	0.0473	1.4100	−1.3946	0.2735	3.6560	3 (0.8)	11.0 (2.9)	0.268 (0.074 –0.968)	**0.0444**	>0.999	0.7473
A1-B8-DR3	0.062	0.053	0.567	6.644	0.0154	0.3796	5.8750	−0.7687	0.2981	3.3545	14 (1.9)	47.0 (6.2)	0.285 (0.155 –0.522)	**0.0001**	**0.0042**	**0.0014**
A2-B7-DR15	0.022	0.016	0.379	3.852	0.0013	0.0375	1.3147	−2.5375	0.2387	4.1886	4 (0.5)	16.8 (2.2)	0.235 (0.078 –0.702)	**0.0095**	0.8074	0.4546
A2-B60-DR13	0.011	0.008	0.236	3.272	−0.0010	−0.4303	0.5697	−2.7027	0.1201	8.3246	1 (0.1)	8.3 (1.1)	0.119 (0.015 –0.949)	**0.0445**	>0.999	0.8883
A2-B62-DR4	0.018	0.013	0.258	3.229	0.0012	0.1015	1.8800	−1.3488	0.1458	6.8582	2 (0.3)	13.7 (1.8)	0.144 (0.032 –0.635)	**0.0105**	0.8827	0.5308
A3-B7-DR15	0.034	0.025	0.372	3.802	0.0147	0.2638	3.2137	−0.5886	0.6192	1.6149	16 (2.1)	25.8 (3.4)	0.611 (0.325 –1.149)	0.1264	>0.999	0.8766
A3-B35-DR1	0.017	0.014	0.399	5.029	0.0014	0.0572	1.5326	−3.4965	0.2351	4.2538	3 (0.4)	12.8 (1.7)	0.232 (0.066 –0.819)	**0.0232**	>0.999	0.7292
A29-B44-DR7	0.009	0.007	0.481	5.383	−0.0003	−1.0000	0.0000	−5.3834	0.0000	–	0 (0.0)	6.6 (0.9)	0.070 (0.006 –0.769)^¶^	**0.0297**	>0.999	0.8700
A24-B8-DR3	0.001	−0.011	−0.929	0.071	0.0093	0.5321	7.8333	7.7618	12.228	0.0818	8 (1.1)	0.7 (0.1)	12.35 (0.991 –153.8)	0.0508	>0.999	0.9915
All other A3-triplotypes	0.019										45 (3.0)	14.3 (1.9)	3.293 (1.799 –6.027)	**0.0001**	**0.0096**	**0.0114**
A3-B18	0.004										11 (0.7)	2.9 (0.4)	3.831 (1.047 –14.02)	**0.0424**	>0.999	0.9563
A3-B44	0.006										12 (0.8)	4.4 (0.6)	2.751 (0.919 –8.235)	0.0705	>0.999	0.9598
A3-B51	0.005										10 (0.7)	3.4 (0.5)	2.962 (0.862 –10.17)	0.0846	>0.999	0.9738
A3-B8	0.005										12 (0.8)	3.5 (0.5)	3.427 (1.043 –11.26)	**0.0424**	>0.999	0.9472
A1-B51 and A3-B51	0.007										16 (1.1)	5.6 (0.7)	2.916 (1.105 –7.692)	**0.0306**	>0.999	0.8913

^|^ Frequencies in healthy blood donors according to Müller et al. (23); D_ab_ = f_ab_–f_a_f_b_, absolute linkage disequilibrium of antigens a and b; D_ab (rel)_, relative linkage disequilibrium of antigens a and b; ^|^ Frequencies in this cohort; RR _diplotype carrier_, relative risk for HNSCC according to ratio of HNSCC versus healthy adults in diplotype carriers; RR _diplotype non-carrier_, relative risk for HNSCC according to ratio of HNSCC versus healthy adults in diplotype non-carriers; OR (95% CI), odds ratio (95% confidence interval); ^†^ p-value from Pearson’s Chi-square (χ^2^) test; ^††^ Benjamini–Hochberg-corrected p-value from χ^2^ tests (25); **^‡^** False-positive reporting probability (FPRP) according to Wacholder et al., 2004 (26); ^¶^ odds ratio corrected according to Cox by adding 1 to each cell preventing division by zero. Significant *p* values < 0.05 shown bold.

As the frequency of particular HLA score defining traits for HNSCC was not substantially different in our cohort, for instance, B51 (11, 12), we analyzed its predictive value for PFS in our cohort of HPV-driven HNSCC. However, this HLA score (12) was not predictive for PFS as we detected neither clear separation of survival curves for various groups based on HLA score mean, median, quartiles, or other cutoffs.

Of special interest would be if antigens deviating in frequency versus healthy controls or among HPV-driven HNSCC are linked to different PFSs. However, according to Kaplan–Meier plots and log-rank tests, only B51 that was found in (insignificantly) increased frequency (OR 1.362, 95% CI 0.595– 3.115) was the only antigen with prevalence above 5% with significant impact on outcome (*p* = 0.022 according to log-rank test), and B51 carriers had impaired PFS (median PFS 9.8, 95% CI 0.0 –25.4 months versus 67.9, 95% CI 44.1– 91.8 months). All other HLA traits defining the HLA score were not linked to altered PFS. However, also carriers of B12C, an HLA-B-associated cross-reactive epitope group [CREG; (32–34)] had significantly reduced PFS (*p* = 0.009).

Consequently, we used multivariate Cox proportional hazard regression to identify independent predictors for PFS in HPV-driven HNSCC by considering well-known lifestyle-associated risk factors for development and outcome of HNSCC but also clinical characteristics (11, 12, 16, 18, 19) including localization of the primary lesion.

**Table 3** shows the 13 independent predictors (*Pi*) building the final Cox proportional hazard regression model for PFS extracted via the stepwise-forward likelihood ratio selection method. The final model consists of five clinical and eight genetic covariates of which only one clinical *Pi* but six HLA traits remained significant *Pi* by themselves according to internal validation by bootstrapping applying 1,000 iterations according to TRIPOD recommendations (29). Shown for all 13 *Pi* are the number of events out of 44 PFS events in total accompanied by the percentage of patients having the characteristic and experiencing an event, the *p*-value from univariate Cox regression analysis, the HR, and 2-sided 95% confidence interval (95% CI).

**Table 3:** Independent predictors (*Pi*) building the multivariate Cox proportional hazard regression model (21, 22) for progression-free survival data-driven extracted via the stepwise-forward likelihood ratio selection method of covariates (29–31) among HPV-driven head and neck squamous cell carcinoma.

Covariate	Ref.	Characteristic	PFS events n (%)	*p*-value univariate	HR	(95% CI)		*p*-value multi-variate	Loss in *χ* ^2^ for deleted term	*p*-value for deleted term	*p-*value for bootstrap	Natural logarithm *ln*_HR_
Alcohol (g/day)	<60	> 60	14 (73.7)	**0.0002**	3.839	(1.858 –7.931)	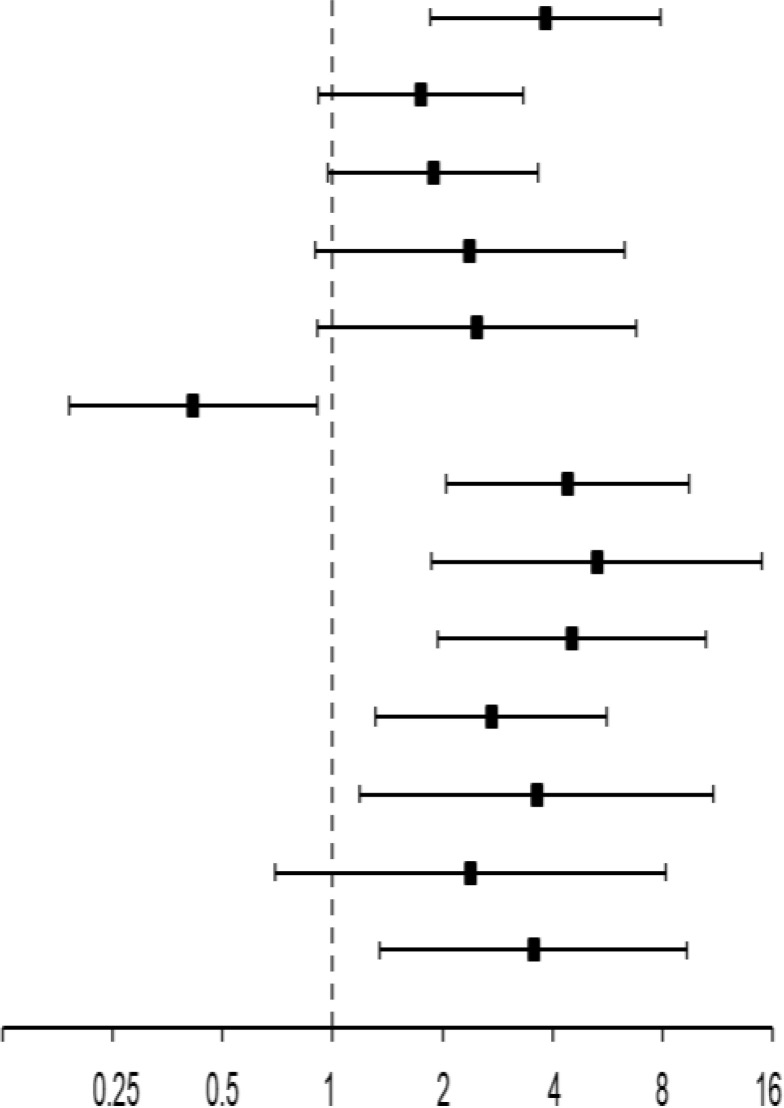	**0.0003**	11.955	**0.0005**	**0.0040**	1.345
Localization	OPSCC	HNSCC	20 (54.1)	0.0743	1.747	(0.917 –3.327)		0.0897	2.896	0.0888	0.1508	0.558
T category	Other T	T4	16 (61.5)	**0.0457**	1.884	(0.969 –3.664)		0.0619	3.323	0.0683	0.1209	0.633
ENE	N0 or ENE-	ENE+	18 (52.9)	0.2642	2.374	(0.894 –6.304)		0.0826	2.888	0.0892	0.1928	0.865
Therapy	Op+PORCT	Other	26 (41.9)	0.6672	2.488	(0.910 –6.803)		0.0759	3.064	0.0800	0.1818	0.911
A1C	Non-carrier	Carrier	29 (44.6)	0.3678	0.415	(0.190 –0.903)		**0.0266**	4.896	**0.0269**	0.0579	-0.880
B12C	Non-carrier	Carrier	28 (62.2)	**0.0075**	4.403	(2.049 –9.463)		**0.0001**	15.488	**0.0001**	**0.0040**	1.482
A11	Non-carrier	Carrier	6 (60.0)	0.1416	5.295	(1.870 –14.99)		**0.0017**	8.076	**0.0045**	**0.0050**	1.667
B51	Non-carrier	Carrier	9 (64.3)	**0.0220**	4.522	(1.939 –10.54)		**0.0005**	10.175	**0.0014**	**0.0050**	1.509
DR15	Non-carrier	Carrier	13 (52.0)	0.2515	2.719	(1.310 –5.642)		**0.0072**	6.867	**0.0088**	**0.0340**	1.000
DQB1 hom	Non-carrier	Carrier	9 (50.0)	0.5926	3.621	(1.189 –11.03)		**0.0235**	4.350	**0.0037**	**0.0230**	1.287
A3-B18	Non-carrier	Carrier	4 (57.1)	0.7324	2.387	(0.697 –8.177)		0.1662	1.693	0.1931	0.1648	0.870
A1-B8	Non-carrier	Carrier	7 (53.8)	0.8396	3.548	(1.347 –9.345)		**0.0104**	5.689	**0.0171**	**0.0400**	1.266

Shown are the number of events out of 44 events associated with each Pi in total and the percentage of patients sharing the characteristic experiencing an event, the p-value from univariate analysis, the hazard ratio (HR), and two-sided 95% confidence interval (95% CI) accompanied by the respective p-value as well as the loss in χ^2^ and the associated p-value for omitting the covariate (for the deleted term), the p-value from bootstrapping applying 1,000 iterations (29), and the natural logarithm (ln) of the HR used for summarizing the individual patient’s risk according to his individual characteristics. Significant p-values are shown in bold.

**Figure 2:**
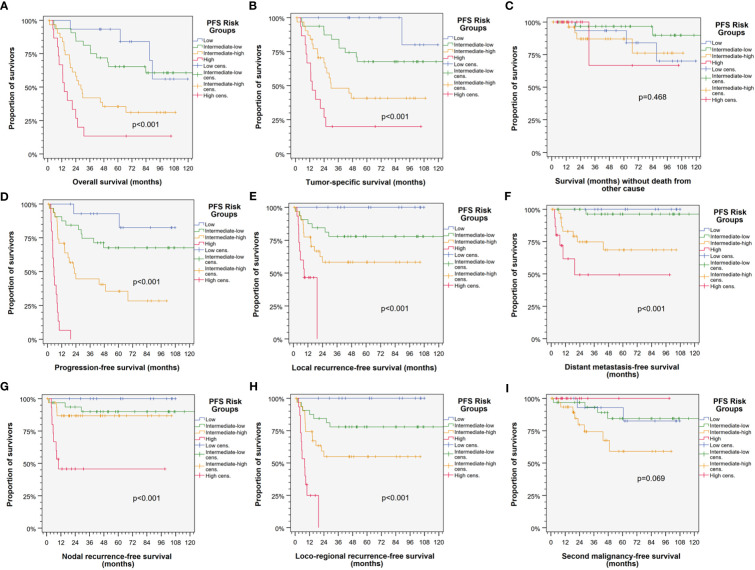
Kaplan–Meier cumulative survival plots (27) depicting well-separated survival curves for the four PFS risk groups according to categorization of the individual patient belonging to either the low (blue), intermediate-low (green), intermediate-high (orange), or high risk group (red) based on the sum of natural logarithms of the hazard ratios of independent predictors in the multivariate Cox proportional hazard regression model (21, 22) reveal good prognostication of HNSCC-specific outcome. **(A)** Progression-free survival (PFS); **(B)** tumor-specific survival (TSS); **(C)** overall survival (OS); **(D)** locoregional recurrence-free survival (LRRFS); **(E)** local recurrence-free survival (LRFS); **(F)** survival without death form other cause censoring HNSCC-related death; **(G)** distant metastasis-free survival (DMFS); **(H)** nodal recurrence-free survival (NRFS); and **(I)** second malignancy-free survival censoring all events from squamous cell carcinoma(s). p-value s shown are from log-rank tests.

Split according to the 16^th^, 50^th^ and 84^th^ percentile of the sum of *ln _HR_* for the 13 *Pi*, the patients were categorized into low, intermediate-low, intermediate-high, and high risk patients showing significant different outcome regarding PFS, TSS, OS, LRFS, NRFS, LRRFS, and DMFS (**Figure 2**). The respective Kaplan–Meier cumulative survival plots for these outcome measures demonstrated early curve separation and good discrimination (all with *p* < 0.001). However, death from other causes and SMFS was not significantly different among PFS risk groups.

As revealed by individual Kaplan–Meier plots for PFS of the four PFS risk groups (**Supplementary Figure S1**), HPV-driven HNSCC differed not systematically in PFS according to localization of the primary tumor (all *p* > 0.38).

**Table 4** shows that 6 of the 8 HLA traits (antigens and haplotypes) with significant impact on outcome (progression-free and overall survival) had equal frequencies among the 94 patients with HPV-driven OPSCC *versus* those from other localization. The only exceptions were A11 (*p* = 0.036) and the haplotype A3-B18 (*p* = 0.027). However, correction for multiple comparisons according to Benjamini–Hochberg (25) results in *p* > 0.2 for each HLA trait between localization groups, indicating impossibility to infer the systematic impact of the localization on particular genetic differences according to our data.

**Table 4:** Distribution of antigen and haplotype frequencies with significant impact on outcome (progression-free and overall survival) among *N* = 94 patients with HPV-driven (HPV-DNA+ RNA+ or HPV-DNA+ p16+) head and neck squamous cell carcinoma with primary in the oropharynx (OPSCC; *n* = 57, 60.6%) or outside the oropharynx (other; *n* = 37, 39.4%).

HLA s	Total		OPSCC		Other HNSCC			
	*n*	(%)	*n*	(%)	*n*	(%)	OR (95% CI)	*p-*value ^†^
A1C								
Non-carrier	29	(30.9)	18	(31.6)	11	(29.7)	Ref. (0.346 –2.889)	0.850
Carrier	65	(69.1)	39	(68.4)	26	(70.3)	0.917 (0.373 –2.253)	
B12C								
Non-carrier	49	(52.1)	31	(54.4)	18	(48.6)	Ref. (0.440 –2.274)	0.586
Carrier	45	(47.9)	26	(45.6)	19	(51.4)	0.795 (0.347 –1.820)	
A11								
Non-carrier	84	(89.4)	54	(94.7)	30	(81.1)	Ref. (0.532 –1.880)	**0.036**
Carrier	10	(10.6)	3	(5.3)	7	(18.9)	0.238 (0.057 –0.989)	
B51								
Non-carrier	80	(85.1)	48	(84.2)	32	(86.5)	Ref. (0.531 –1.882)	0.762
Carrier	14	(14.9)	9	(15.8)	5	(13.5)	1.200 (0.368 –3.910)	
A1-B8								
Non-carrier	81	(86.2)	48	(84.2)	33	(89.2)	Ref. (0.534 –1.872)	0.495
Carrier	13	(13.8)	9	(15.8)	4	(10.8)	1.547 (0.439 –5.445)	
A3-B18								
Non-carrier	87	(92.6)	50	(87.7)	37	(100.0)	Ref. (0.548 –1.824)	**0.027**
Carrier	7	(7.4)	7	(12.3)	0	–	11.14 (0.617 –201.2) ^#^	
DR15								
Non-carrier	69	(73.4)	44	(77.2)	25	(67.6)	Ref. (0.499 –2.002)	0.302
Carrier	25	(26.6)	13	(22.8)	12	(32.4)	0.616 (0.244 –1.553)	
DQB1 homozygosity								
Non-carrier or heterozygous	85	(90.4)	52	(91.2)	33	(89.2)	Ref. (0.540 –1.853)	0.743
DQB1 homozygous	9	(9.6)	5	(8.8)	4	(10.8)	0.793 (0.199 –3.170)	
HLA risk groups								
Low risk	18	(19.1)	12	(21.1)	6	(16.2)	Ref. (0.250 –3.999)	0.891
Intermediate-low risk	29	(30.9)	18	(31.6)	11	(29.7)	0.818 (0.238 –2.811)	
Intermediate-high risk	34	(36.2)	19	(33.3)	15	(40.5)	0.633 (0.192 –2.084)	
High risk	13	(13.8)	8	(14.0)	5	(13.5)	0.800 (0.181 –3.536)	
Clinical risk groups								
Low risk	28	(29.8)	28	(49.1)	0	–	Ref. (0.019 –52.16) ^#^	**< 0.001**
Intermediate-low risk	25	(26.6)	1	(1.8)	24	(64.9)	0.001 (0.000 –0.023)	
Intermediate-high risk	28	(29.8)	22	(38.6)	6	(16.2)	0.064 (0.003 –1.215)	
High risk	13	(13.8)	6	(10.5)	7	(18.9)	0.015 (0.001 –0.302)	
PFS risk groups (HLA + clinical)								
Low risk	15	(16.0)	14	(24.6)	1	(2.7)	Ref. (0.057 –17.62)	**0.037**
Intermediate-low risk	32	(34.0)	18	(31.6)	14	(37.8)	0.092 (0.011 –0.785)	
Intermediate-high risk	32	(34.0)	18	(31.6)	14	(37.8)	0.092 (0.011 –0.785)	
High risk	15	(16.0)	7	(12.3)	8	(21.6)	0.063 (0.006 –0.604)	

^†^ p-values from Pearson’s Chi-square (χ^2^) tests for contingency tables; # odds ratio corrected according to Cox by adding 1 to each cell preventing division by zero; significant p-values < 0.05 highlighted in bold.

## Discussion

Our study revealed strong similarities of HPV-driven HNSCC independent from localization either in the oropharynx or in other localizations in the head and neck region. Despite significant differences in some clinical characteristics appearing to be strong modifiers of locoregional metastasis at the time of diagnosis and differences regarding N categories according to the 7th (14) and, even more pronounced, the 8th edition of TNM staging (20), HLA antigen and haplotype frequencies related to localization were similar. Moreover, and despite a trend towards higher risk for relapse in HPV-driven HNSCC arising outside the oropharynx (HR 1.747, 95% CI 0.917 to 3.327; *p* = 0.0897), the impact of those HLA traits representing *Pi* (**Table 3**) on outcome was not dependent on the primary’s localization as revealed by the newly developed PFS risk score (**Figure 2**, **Supplementary Figure S1**).

**Figure 3:**
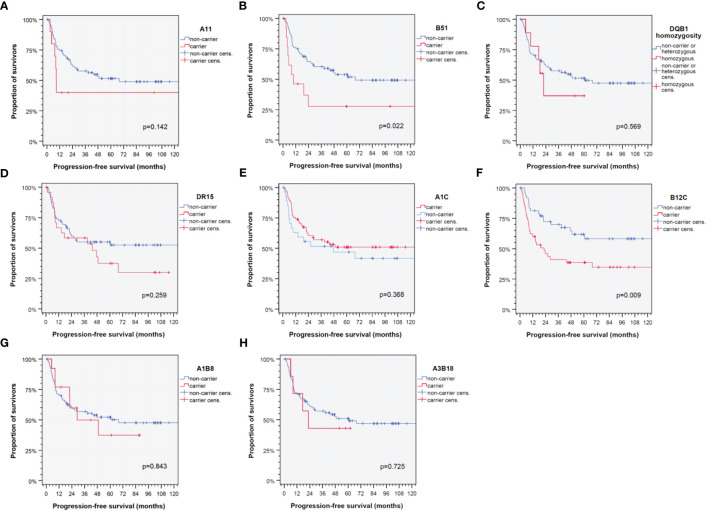
Kaplan–Meier cumulative survival plots (27) for HLA traits that are found to be significant independent predictors of progression-free survival of HPV-driven HNSCC as detected in the multivariate Cox proportional hazard regression model (21, 22). **(A)** A11; **(B)** B51; **(C)** DQB1 homozygosity; **(D)** DR15; **(E)** cross-reactive epitope group A1C; **(F)** cross-reactive epitope group B12C; **(G)** diplotype A1-B8; and **(H)** diplotype A3-B18. *p*-values shown are from log-rank tests.

HPV-driven HNSCC shares deviating HLA antigen and haplotype frequencies compared to other (pre-dominantly HPV-negative) HNSCCs and healthy blood donors (**Table 2**). As we were interested in the applicability of the HLA score, we first analyzed frequencies of particular HLA traits. Here, we confirm an enrichment in B51 carriers compared to healthy blood donors recently reported (35). However, compared to the training cohort and independent validation cohort of our earlier investigation (11, 12), HLA-C homozygosity was the only HLA trait demonstrating a lower frequency in HPV-driven HNSCC (20.2% versus 30.3%; *p* = 0.093), whereas all other frequencies of particular HLA traits were not different (all *p* > 0.22). We detected, however, significant different haplotype frequencies and linkage disequilibrium relationships compared to subjects without malignancy (**Table 2**) (23). While we found strong protective effects of the most common haplotypes in Caucasian German blood donors (23) confirming, e.g., reports about the protective effects of A1-B8 on other malignant diseases (6), rare haplotypes (4, 9, 23) were found in significantly higher frequency (**Table 2**). Among those rarely detected in healthy volunteers and in HPV-driven HNSCCs, more prevalent haplotypes (4, 9, 23) that were found to be enriched here were A3 triplotypes. While A3-B7-DR15 and A3-B35-DR1, triplotypes that are among the top 20 haplotypes in blood donors (23), were found at a lower frequency, the sum of all other possible A3 triplotypes was significantly increased (OR 3.293, 95% CI 1.799 –6.027; *p* = 0.0001) and remained significant even after correction for multiple testing (*p* = 0.0096 after Benjamini–Hochberg correction) with a low FPRP according to Wacholder et al. (26). As the frequencies of these haplotypes are nevertheless rather low, one might expect OR for individual A3 haplotypes not to constitute significant risk factors for the development and outcome of an HPV-driven disease. However, A3-B8 and A3-B18 are observed at a significantly higher frequency and also linked to poor outcome in HPV-driven HNSCC (**Figure 3**). In this respect, the increased OR translates also into an increased HR arguing for a causal relationship according to several converging lines of evidence.

In this respect, A3-B18 is an independent predictor of poor outcome (**Table 3**). In the light of an earlier report about rather impaired response and outcome of oncologic patients carrying A3 when treated with immune-checkpoint inhibitors, anti-PD-1 and/or anti-CTLA-4 antibodies (36), we wonder if this observation could rather reflect reduced immune competence based on disrupted haplotypes (and occurrence with HLA-B and -DR antigens in unfavorable combinations). Moreover, as we show that the linkage disequilibrium of common haplotypes is reduced and their frequency in HPV-driven HNSCC patients is significantly lower than among other HNSCC and healthy blood donors (compare (23) and **Table 2**, **Supplementary Table S1**), those subjects protected by carrying a favorable haplotype might indeed have a lower risk to develop the disease and consequently might have led to an enrichment of unfavorable haplotypes in our cohort. Therefore, this explanation together with the detrimental impact of, for instance, B51 itself could have contributed to increased odds and HRs observed.

Consecutively, we tried to answer the question if the HLA score for HNSCC would be predictive for PFS in our cohort of HPV-driven HNSCC. However, this was not the case as we detected neither a clear separation of survival groups based on HLA score (12) quartiles, median, or other cutoffs (data not shown). Therefore, we first assessed differences in the distribution of clinical and epidemiologic factors related to localization and their impact on outcome.

Although similar distribution of T categories among localization groups was observed at diagnosis, cases with primary within the oropharynx had locoregional metastases in higher frequency and higher numbers of disease-positive neck nodes. The relevance of the localization for locoregional metastasis detected confirms the well-known higher frequency of neck nodes in OPSCC and was found to be independent from the HPV status as detected in another cohort (37). However, even though differences in the number of positive neck nodes and deviating frequency of various N categories were noticed, the distribution of ENE-negative and ENE-positive neck nodes among patients with locoregional metastases was similar in HPV-driven OPSCC and other HNSCC (**Table 1**) and in both groups linked to impaired outcome (**Figure 1E**, **Table 4**). In this regard, it was no surprise that, in contrast to ENE (HR 2.374, 95% CI 0.894 to 6.304; *p* = 0.0826), the number of positive neck nodes and the N category were not found to be independent predictors of PFS. This confirms earlier findings (18, 19). However, significant differences in T categories according to the 8th edition of TNM staging (20) appear to be related only to the deviating categorization of p16+ OPSCC (for which T4 are no longer subdivided into T4a and T4b) but not to differing biology or other meaningful differences. The same appears to be true but even more misleading regarding discrimination of N categories according to the 8th edition. The 8th edition categorizes nodal metastasis in p16+ OPSCC in extremely discrepant ways (20). The N categories in ENE-negative HNSCC are based on the maximum diameter of the largest node and sub-classify ipsilateral versus contra- or bilateral positive nodes. Considering the increased risk attributable to ENE, N categories are heightened accordingly (N2a for a single node < 3 cm and N3b for all other cases). In contrast, ENE is disregarded in N categories of p16+ OPSCC by only counting the number of disease-positive resected nodes (20). This contrast was based on the ICON-S study (38) that showed the outcome in HPV-related (p16+ OPSCC but not essentially HPV-driven) cases treated via radiotherapy or cisplatin-based radio-chemotherapy deviating from the expected outcome according to the 7th edition of TNM staging (14). However, not considering unique features appears to be artificial and not substantiated by deviating biology or the different outcome of HPV-driven HNSCC either in the oropharynx (p16+ OPSCC) or in other head and neck regions (and therefore also not helpful in decision-making for treatment in daily routine) (39).

The so-called “classical risk factors” for HNSCC development alcohol consumption and tobacco smoking history (16, 18, 19, 37) were present in nearly the same frequency in localization groups (all *p* > 0.33). However, Kaplan–Meier estimates showed a significant impact on PFS for tobacco smoking (< 20 pack years versus ≥ 20 pack years; *p* = 0.014) and daily alcohol consumption (never to < 60 g versus ≥ 60g; *p* < 0.0002). In our previous work (19), daily alcohol consumption > 30 g was identified as an independent risk factor (*Pi*) for disease-free survival (DFS) with HR 2.8 (95% CI 1.1 –7.1), *p* = 0.030, in multivariate analysis and stable *Pi* after 1,000 bootstrap iterations (*p* = 0.045). Alcohol > 30 g/day was a *Pi* for PFS with HR 2.63 (95% CI 1.05– 6.58), *p* = 0.0395, in multivariate analysis and stable *Pi* after 1,000 bootstrap iterations (*p* = 0.045). As a previous study only included p16-positive local advanced oropharyngeal SCC undergoing upfront surgery followed by risk factor adapted adjuvant therapy, the present paper represents a validation of this report about the impact of high-level alcohol consumption on outcome in HPV-driven HNSCC from an independent cohort. Therefore, the detrimental impact of alcohol on outcome seems to be a valid finding. According to heterogeneity in distribution of other clinical characteristics, T4 category (14, 20) was the only other significant risk factor for PFS emerging (*p* = 0.046).

Consequently, we analyzed the impact of multiple covariates in multivariate Cox proportional hazard regression models (21, 22). First, clinical risk factors known for development or recurrence of HNSCC were analyzed to identify their impact on PFS. Consecutively, we iteratively included all available clinical, epidemiological, and genetic information and were finally able to build a multivariate Cox proportional hazard regression model for PFS via data-driven automatic stepwise forward selection of covariates among p16-positive HR-HPV DNA-positive HNSCC as recommended (30, 31). **Table 4** shows the 13 independent predictors of PFS, namely, 5 clinical and 8 genetic covariates. Interestingly, only two of the HLA traits defining the HLA score for predominantly HPV-negative HNSCC are included in the new model (**Figure 2**; **Table 4**). These are B51 and A1-B8. It is important to note that B51 had a somewhat lower impact on PFS (HR 4.52, 95% CI 1.94– 10.5; *p* = 0.0005) within the 95% CI (HR 9.28, 95% CI 2.27– 37.9) of the prior investigations (11, 12), but A1-B8 demonstrated an opposite impact on PFS in HPV-driven HNSCC (HR 3.55, 95% CI 1.35– 9.35; *p* = 0.010). The other HLA antigens included in the model are DR15 and DQB1 homozygosity besides A11 and two CREGs (32–34), A1C and B12C. DR15 was described as a risk factor for cervix carcinoma in numerous investigations and also detected in HPV-related OPSCC (35), and according to the strong linkage disequilibrium of DR15 and DQ6 (haplotype DR15-DQ6 according to HLA-DRB1*15-DQB1*06), DQB1*06 that is an independent predictor for PFS in HNSCC may have missed inclusion in the new model due to inclusion of DR15 that achieved a higher level of significance and therefore was data-dependent and automatically extracted to be included in the model, which is the recommended approach (29–31). However, DQB1 homozygosity independently reduces PFS.

A1C and B12C are CREGs defining shared epitopes able to bind to activating and stimulating NK cell receptors. The antigens A1, A3, A11, A29, A30, A31, A36, and A80 share the epitope defining the CREG A1C (32, 33). Obviously, A1C was linked to a superior outcome in these HLA-A alleles, with A11 being the only exception. Interestingly, A3 and A11 share 149A+150A+151H, whereas the common sequence of A1 is 149A+150V+151H and associated with stronger peptide binding and immune responses (34). There is accumulating knowledge about A1 and A3 alleles sharing the reduced binding capability of HPV16 E7-derived peptides (8, 40–43). A3-B18 was a haplotype detected in significantly increased frequency and strongly reducing PFS of carriers. Besides the implication of A3 in the reduced ability to eradicate tumors in response to immune-checkpoint blockade (36), B18 may essentially contribute to impaired response to neoantigens including HPV-derived peptides. B18 is known to be the HLA-B member with the lowest binding capability among members of the HLA-B44 supertype (35). B12C is a CREG shared by B12 (B44 and B45), B13, B21 (B49 and B50), B37, B40 (B60 and B61), B41, and B47 (32–34). B12C is characterized by the amino acid threonine at position 41 (41T) (34). Among B12C, several disease-associated alleles/antigens are gathered, for instance, B13 and B37 (psoriasis), B40 (acute lymphatic leukemia), B41 (hepatitis), and B47 (adrenogenital syndrome), and there is a strong overlap with the HLA-B44 supertype characterized by particular peptide binding properties (8). The B and F pockets’ binding specificities for acidic and aromatic/aliphatic/hydrophobic amino acid residues, respectively (8), may be involved in a rather reduced binding capability of otherwise immunogenic HPV16 E6 and E7 peptides (35).

A11, also an A1C CREG member (32–34), was reported to be involved in several diseases, and conflicting results of involvement of A11 in chronic periodontitis were reported for German patients and HPV18-associated tumors (44, 45). Antigenic E6 peptides derived from HPV18 were reported to be potential candidates for the treatment of HPV18-associated tumors in HLA-A11-positive populations as they identified two HLA-A11-restricted epitopes derived from HPV18 E6 oncoprotein for CD8^+^ cytotoxic T cells (45). Especially because we found A11 to be an independent risk factor for reduced PFS in HPV-driven HNSCC, their findings could be a promising approach for future vaccination or even therapy of HPV-associated tumors in A11-positive patients. We therefore would also like to recommend A11 presentable peptides to be included into a vaccine designed according to Mühlenbruch et al. (35).

Our study has limitations. First, we included HPV-driven HNSCC defined by p16 positivity and the presence of high-risk HPV-DNA positivity, and not all patients were confirmed to be transcriptionally active according to the presence of E6*I (37) or anti-E6 serology or according to HPV sero-pattern positivity (46, 47) to definitively verify the status “HPV-driven”. Second, the number of patients with HPV-driven HNSCC outside the oropharynx is 37 and therefore small. However, because HPV-driven HNSCCs rarely develop outside the oropharynx, it took a long time to gather even this cohort. Third, we did not perform next-generation sequencing of HLA for exact high-resolution typing and focused on antigens and haplotypes according to serologic markers. However, as the level of interaction between immune cells including tumor-infiltrating T and NK cells and cancer cells is based on protein–protein interaction, this may also be seen as a strength. Fourth, we could only estimate haplotypes as we were not able to execute HLA typing on siblings, parents, etc. This, of course, has reduced the power to detect deviations from expected haplotype frequencies. Therefore, despite our findings that HPV-driven HNSCCs share an enrichment in rather rare haplotypes, and common haplotypes are rather protective for developing HPV-driven HNSCC, we surely have underestimated the deviations. Fifth, despite the internal validation of independent predictors (*Pi*) for PFS applying bootstrapping (29) and detecting good discrimination of the four PFS risk groups also according to other HNSCC-related outcome measures like TSS, LRFS, NRFS, LRRFS, and DMFS, the score could represent an overfitting to our cohort’s characteristics, and therefore can be seen only as provisional before validation in an independent cohort. This will be the next step.

## Conclusion

Independent from localization in the oropharynx or other localizations in the head and neck region, HPV-driven HNSCCs share common risk factors and particular HLA antigens and haplotypes associated with the development of HNSCC and relapse after therapy with a curative intent. Based on the multivariate Cox proportional hazard regression model for PFS, 13 independent predictors were identified. Clinical risk factors linked to impaired PFS include daily alcohol consumption ≥ 60 g, localization of the primary outside the oropharynx, T4 category, extranodal extension of neck nodes, and treatment other than upfront surgery followed by cisplatin-based post-operative radio-chemotherapy. However, only daily alcohol consumption ≥ 60 g remained a significant *Pi* according to internal validation by bootstrapping. In sharp contrast, six out of eight HLA traits were significant predictors of PFS even after applying the bootstrap. Calculating the sum of natural logarithm for the individual patient allowed for the development of a new prognostication model for HPV-driven HNSCC that demonstrates good prognostication of outcome for PFS risk groups (low, intermediate-low, intermediate-high, and high) according to PFS, TSS, OS, LRFS, NRFS, LRRFS, and DMFS. A validation of the PFS risk model in an independent cohort of HPV-driven HNSCC is required.

## Data availability statement

The raw data supporting the conclusions of this article will be made available by the authors, without undue reservation.

## Ethics statement

The studies involving humans were approved by The Institutional Human Ethics Committee of the University Leipzig (votes 201-10-12072010, 202-10-12072010, and 341-15-09102015). The studies were conducted in accordance with the local legislation and institutional requirements. The participants provided their written informed consent to participate in this study.

## Author contributions

Conceptualization, GW. Methodology, GW, NV, CL, and RL. Validation, GW, NV, and TW. Formal analysis, GW, and NV. Investigation, GW, and NV. Resources, GW, CL, RL, AD, VZ, and SW. Data curation, GW, and NV. Writing—original draft preparation, GW, NV, and TW. Writing— review and editing, all authors. Visualization, GW, NV and TW. Supervision, GW, CL, and SW. Project administration, GW. Funding acquisition, GW, and AD. All authors contributed to the article and approved the submitted version.
