# Supplementary material for: Outcome differences in HPV-driven head and neck squamous cell carcinoma attributable to altered human leukocyte antigen frequencies
Source: Front Oncol. 2023 Dec 21;13:1212454. doi: 10.3389/fonc.2023.1212454 (PMC10772155; doi:10.3389/fonc.2023.1212454)
Supplement: Supplementary file 1 [file DataSheet_1.pdf]

Table S1. Frequencies of top 20 triplotypes in a large German sample of healthy blood donors [21] and HPV-driven head and neck squamous cell carcinoma (HNSCC; this study) accompanied by linkage disequilibrium, odds ratio (OR) and 95% confidence interval (95% CI) together with the 2-sided *P* value (*P* value †), Benjamini-Hochberg corrected *P* value (*P* value ††) [23] and false-positive reporting probability (FPRP) [24] and the effect on development of HPV-driven HNSCC according to detected genetic association in our cohort.

	$f_{observed}$ |	diplotype frequency f_{ab}	allele frequency f_a	allele frequency f_b	$D_{ab} = f_{ab} - f_a f_b$ | |	OR (95% CI)	*P* value †	*P* value ††	FPRP-level ‡	Effect
A1-B8-DR3	0.062	0.019	0.085	0.037	0.015	0.285 (0.155 - 0.522)	**0.00004**	**0.0042**	0.0014	Protective
A1-B57-DR7	0.009	0.001	0.106	0.008	0.0005	0.143 (0.017 - 1.163)	0.0689		0.9365	Protective §
A2-B7-DR15	0.022	0.005	0.138	0.029	0.001	0.235 (0.078 - 0.702)	**0.0095**	0.8074	0.4546	Protective
A2-B8-DR3	0.007	0.005	0.085	0.016	0.004	0.801 (0.214 - 2.998)	0.7420		0.9955	
A2-B13-DR7	0.009	0.004	0.106	0.013	0.003	0.415 (0.108 - 1.604)	0.2025		0.9767	
A2-B44-DR4	0.014	0.011	0.133	0.029	0.007	0.775 (0.306 - 1.964)	0.5914		0.9884	
A2-B44-DR11	0.007	0.001	0.090	0.029	-0.001	0.190 (0.022 - 1.614)	0.1281		0.9733	
A2-B44-DR13	0.006	0.001	0.079	0.029	-0.001	0.208 (0.024 - 1.796)	0.1533		0.9795	
A2-B57-DR7	0.008	0.009	0.106	0.021	0.007	1.128 (0.381 - 3.339)	0.8276		0.9949	
A2-B60-DR13	0.011	0.001	0.079	0.029	-0.001	0.119 (0.015 - 0.949)	**0.0445**	> 0.999	0.8883	Protective
A2-B62-DR4	0.018	0.003	0.133	0.011	0.001	0.144 (0.032 - 0.635)	**0.0105**	0.8827	0.5307	Protective
A2-B62-DR13	0.008	0.003	0.079	0.011	0.002	0.332 (0.067 - 1.650)	0.1777		0.9778	
A3-B7-DR15	0.034	0.021	0.138	0.048	0.015	0.611 (0.325 - 1.149)	0.1264	> 0.999	0.8766	
A3-B35-DR1	0.017	0.004	0.122	0.021	0.001	0.232 (0.066 - 0.819)	**0.0232**	> 0.999	0.7291	Protective
A11-B35-DR1	0.007	0.003	0.122	0.013	0.001	0.353 (0.070 - 1.779)	0.2072		0.9821	
A23-B44-DR7	0.007	0.003	0.106	0.005	0.002	0.368 (0.072 - 1.867)	0.2275		0.9843	
A24-B7-DR15	0.006	0.004	0.138	0.013	0.002	0.589 (0.141 - 2.461)	0.4676		0.9927	
A25-B18-DR15	0.007	0.007	0.138	0.013	0.005	0.919 (0.272 - 3.109)	0.8921		0.9959	
A29-B44-DR7	0.009	0.000	0.106	0.003	-0.0002	0.070 (0.006 - 0.769)¶	**0.0297**	> 0.999	0.8700	Protective
A30-B13-DR7	0.007	0.004	0.106	0.008	0.003	0.610 (0.145 - 2.577)	0.5016		0.9935	

| Frequencies in healthy blood donors according to Müller et al. [21]; $D_{ab} = f_{ab} – f_a f_b$, absolute linkage disequilibrium of antigens *a* and *b*; *OR* (*95% CI*), *odds ratio* (*95% confidence interval*); † *P* value from *Pearson's* Chi-square (χ^2) test; †† Benjamini-Hochberg-corrected *P* value [23] from χ^2 tests; ‡ *False positive reporting probability* (FPRP) according to Wacholder et al., 2004 [24]; ¶ odds ratio corrected according to Cox by adding 1 to each cell preventing division by zero.

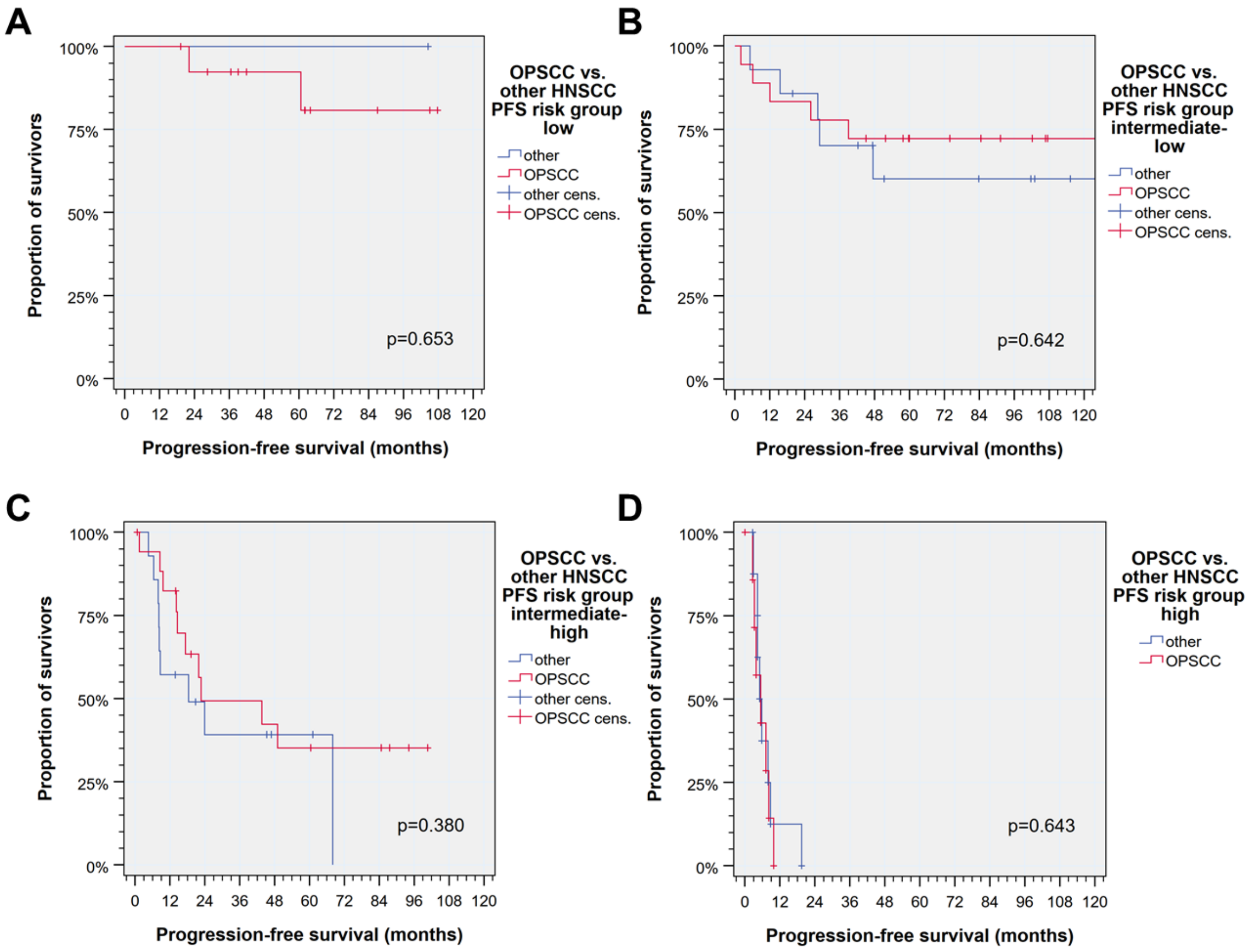

Figure S1. Kaplan-Meier cumulative survival plots [25] for HPV-driven OPSCC *versus* other (HPV-driven HNSCC with primary outside oropharynx) demonstrate insignificant differences in progression-free survival among the individual PFS risk groups according to the newly developed score.
